# The Fancher Case

**Published:** 1879-03-20

**Authors:** 


### *THE FANCHER CASE.*

This is the case of a lady in Brooklyn who, it is authentically stated, has been bed-ridden for about thirteen years, having received an injury by a fall from which she has never recovered. During much of the time she has been unconscious and is extremely emaciated, and it is said has not taken in all these years nourishment sufficient to have kept up an ordinary person a single week.

The most remarkable things are told of her clairvoyant powers, which have astonished intelligent men—clergymen, physicians and others—who have visited and examined the case. Those who are fond of studying nervous and mental phenomena can certainly find in this case matter worthy of profound investigation. The singular powers ascribed to her are said to be in full accord with the occult phenomena of modern spiritualism, and must tend to direct scientific and inquiring minds to the investigation of this mysterious subject, which indeed seems to be pressing itself by steady and persistent encroachments upon the consideration of intelligent and reflective minds.

A correspondent desires to know where the facts of this strange case can be found; to which we reply that they have been published in many of the leading papers North and South. It is said that the most full and satisfactory account of the case, with discussions bearing upon the causes, truth, facts, etc., of the case may be found in *The Religio-Philosophical Journal*, the leading organ of the Spiritualists in the United States, published at Chicago, Ill. This paper is ably edited, and under its late management nas taken a strong stand against deception and imposture in its own ranks, and assumed a higher and more Christian-like stand in connection with this strange and mysterious subject. W.

